# Markov Chain-Based Acute Effect Estimation of Air Pollution on Elder Asthma Hospitalization

**DOI:** 10.1155/2017/2463065

**Published:** 2017-09-24

**Authors:** Li Luo, Fengyi Zhang, Wei Zhang, Lin Sun, Chunyang Li, Debin Huang, Gao Han, Bin Wang

**Affiliations:** ^1^Business School, Sichuan University, Chengdu, Sichuan 610000, China; ^2^Big-Data Center of Biomedicine, West China Hospital, Sichuan University, Chengdu, Sichuan 610000, China; ^3^Medical Insurance Office, West China Hospital, Sichuan University, Chengdu, Sichuan 610000, China; ^4^Chengdu Medical Insurance Administration, Chengdu, Sichuan 610000, China

## Abstract

**Background:**

Asthma caused substantial economic and health care burden and is susceptible to air pollution. Particularly, when it comes to elder asthma patient (older than 65), the phenomenon is more significant. The aim of this study is to investigate the Markov-based acute effects of air pollution on elder asthma hospitalizations, in forms of transition probabilities.

**Methods:**

A retrospective, population-based study design was used to assess temporal patterns in hospitalizations for asthma in a region of Sichuan province, China. Approximately 12 million residents were covered during this period. Relative risk analysis and Markov chain model were employed on daily hospitalization state estimation.

**Results:**

Among PM2.5, PM10, NO_2_, and SO_2_, only SO_2_ was significant. When air pollution is severe, the transition probability from a low-admission state (previous day) to high-admission state (next day) is 35.46%, while it is 20.08% when air pollution is mild. In particular, for female-cold subgroup, the counterparts are 30.06% and 0.01%, respectively.

**Conclusions:**

SO_2_ was a significant risk factor for elder asthma hospitalization. When air pollution worsened, the transition probabilities from each state to high admission states increase dramatically. This phenomenon appeared more evidently, especially in female-cold subgroup (which is in cold season for female admissions). Based on our work, admission amount forecast, asthma intervention, and corresponding healthcare allocation can be done.

## 1. Background

Asthma is a major public health issue in the USA, affecting over 23 million persons [1]. In China, the asthma population has reached 30 million [2]. The prevalence of asthma tends to be higher in urbanized and well-developed areas compared to the developing areas ([3]; Weiland and Pearce, 2004). Moreover, while there has been an overall trend of a decline in prevalence in developed countries (Moorman et al., 2012), there is an increasing trend in developing countries [4]. The prevalence and risk factors of asthma in several metropolitan cities in China including Beijing, Chengdu, and Guangzhou have become comparable to those in other developed countries ([5]; Zhao et al., 2010).

Ambient air pollution has been linked to the development and exacerbation of asthma and its related diseases in Europe, North America, Korea, Japan, and Taiwan ([6]; Jaffe et al., 2003; [7]; Samet et al., 2000; Sunyer et al., 1997; [8]; Yang et al., 2007; [9–11]). Especially, elder asthma patients (older than 65) are considered more fragile to air pollution [7, 12, 13], when compared with other adults, which means that they are associated with a higher probability to consume healthcare resource, due to air pollution. Besides, fewer than 1% of the 500 largest cities in China meet the air quality standards recommended by the World Health Organization, and 7 of these cities are ranked among the 10 most polluted cities in the world [14]. Hence, investigating the association between air pollution and elder admission and analyzing the transition probability in China are of great meaning.

Many works have been done in this field and achieved specific findings. Schouten et al. [8] applied GLM (generalized linear model) to assess the short-term relationship between air pollution and the daily number of emergency hospital admissions for respiratory disease. The results showed that the relation between short-term air pollution and emergency hospital admissions is not always consistent at these rather low levels of daily hospital admissions and of air pollution. Szyszkowicz [15] examined and assessed the potential relations between ED (emergency department) visits for asthma and the concentrations of ambient air pollutants. A generalized linear mixed model was applied and proved the hypothesis that ED visits for asthma are associated with exposure to *O*_3_. Grineski et al. [16] explored the role of race, ethnicity, and insurance status in modifying the effects of air pollution on childrens' asthma hospitalizations in Phoenix, Arizona, by analyzing asthma hospitalization data obtained from the Arizona Department of Health Services. The research suggested that increasing insurance enrollment for all children, specifically Hispanic children, may reduce their disproportionate risk from exceedances of air pollution. Li et al. [17] explored the threshold effects of air pollutants on pediatric asthma, by analyzing Medicaid beneficiary and claims data obtained from the Michigan Data Warehouse of the Michigan Department of Community Health. The study indicates that the associations of SO_2_ and PM2.5 concentrations with asthma emergency department visits and hospitalizations, as well as the estimated PM2.5 (particulate matter not greater than 2.5 mm in aerodynamic diameter) threshold, were fairly consistent across time-series and case-crossover analyses and suggest that effect estimates based on linear models (without thresholds) may underestimate the true risk. Cai et al. [12] applied an overdispersed generalized additive model to investigate the acute effect of air pollution on asthma hospitalization in Shanghai, China, and found that the effects of SO_2_ and NO_2_ were robust after adjustment for PM10. The associations appeared to be more evident in the cold season than in the warm season. Cho et al. [9] applied conditional logistic regression to investigate the short-term effect of ambient air pollution on the risk of asthma. By analyzing the data from medical claims which were reported to the Health Insurance Review and Assessment Service (HIRA), it was indicated that SO_2_, PM10 (particulate matter not greater than 10 mm in aerodynamic diameter), NO_2_, and CO were positively associated with ED visits for asthma.

However, previous studies have serval limitations: (1) Although elder asthma patients were found to be more fragile to air pollution [12], sex and seasonality may be the potential risk factors of elder asthma hospitalization. However, previous work did not take age, sex, and seasonality into consideration simultaneously. (2) Previous works focused only on the overall ratio between the increment of air pollutants and hospitalization, which limited contributions in practice. However, to achieve the distribution of future hospitalization considering air pollution is much more helpful. Powerful tools such as Markov chain should be employed, due to their excellent performances in transition probability estimation [18, 19].

This study aims to investigate the Markov-based acute effects of air pollution on elder asthma hospitalization, which is the key chain of forecasting admission amount, by measuring the acute effects of air pollution on elder asthma admission first. We take sex and seasonality factors into consideration to achieve a systematic research. The Markov model is particularly useful in analyzing risk factors in cohort studies and has been applied successfully to the study of lung cancer, HIV infection [19], and the cost of asthma [18]. The results in terms of relative risk and transition probability curves are practical and easy to understand. Moreover, the Markov assumption is somewhat restrictive where it supposes that the probability of a changing state depends only on the current state and not on previous history of state transitions. We construct Markov chain, which is an effective way to not only describe the association between air pollution and asthma resource demand but also achieve a distribution of demand. This study outputs Markov transition probabilities between asthma admission amount states under different air pollution situations. When combined with air pollution forecast for a continuous period, the Markov chain can infer future distribution of admission amount states for each day. In fact, Deo et al. [20] developed an integrated Markov-based capacity allocation model that incorporates clinical (disease progression) and operational (capacity constraint) aspects for chronic disease. However, their study focused on improving the patients' QALY (quality-adjusted life years) by healthcare resource allocation, without considering the demand varying with air pollution, not to mention making corresponding intervention and then allocating matching healthcare resource. In this study, we apply Markov chain to describe the elder asthma admission evolution process considering air pollution. Based on our work, admission amount forecast, asthma intervention, and corresponding healthcare allocation can be done.

## 2. Method

### 2.1. Data

Our study area covers a region of Sichuan province, China. We obtained inpatient records of asthma hospitalization for adult residents between January 1, 2014, and December 31, 2014 (365 days). Approximately 12 million residents were covered during this period. The main diagnoses of hospital admission were coded according to the International Classification of Diseases, Revision 10 (ICD-10): Asthma (J45.001, J45.005, J45.901, J45.902, and J45.903). The data was also classified by season and sex. Warm season is defined as a period from April to September, and cold season is defined as the rest of time period in a year. Elder person is defined as person older than 65 years.

Daily (24 h) air pollution concentration data including particulate matter less than 2.5 mm in aerodynamic diameter (PM2.5), particulate matter less than 10 mm in aerodynamic diameter (PM10), sulfur dioxide (SO_2_), and nitrogen dioxide (NO_2_), from January 1, 2014, to December 31, 2014, were obtained from the website of the Environmental Monitoring Center (EMC) database. To allow adjustment for the effect of weather on hospital admission, meteorological data (daily min temperature) were obtained from the website of Meteorological Bureau.

### 2.2. Statistical Analysis

Daily asthma hospitalization and air pollution levels were linked by date and therefore could be analyzed with a time-series design. Because daily hospital admission for asthma approximately follows a Poisson distribution [12], we utilized generalized linear Poisson models to estimate the association of asthma hospital admission with air pollution levels. We incorporated the ns functions of min temperature (6 df for the period) to adjust for the potential nonlinear confounding effects of weather conditions [21]. After establishing the basic model, we introduced the air pollutant concentrations into the single-pollutant model one at a time to estimate their associations with asthma hospitalization. We also included the day of the week as an indicator variable in the basic models. We examined the effect of air pollutants with different lag structures from lag0 (current day) to lag0–5 (recent six days). Lag0–5 corresponds to 6-day moving average of pollutant concentration of recent six days in RR (relative risk) analysis. These period models were used for our main analysis, given that single-day lag models may underestimate the cumulative effect of pollutants on hospital admissions [22]. We also conducted season- and sex-specific analysis, and the air pollution effects on asthma hospitalization between subgroups were compared. Unless specified otherwise, the results are presented as the percent change in daily hospital admission for a 10 *μ*g/m^3^ increase in the pollutant concentration and 95% confidence intervals (CIs). In this way, the pollutants which acutely effect elder asthma admission can be confirmed.

For the purpose of healthcare resource allocation and schedule, it is important to measure the association between air pollution and elder asthma admission amounts and make corresponding forecast. Markov chain is a useful way to describe the asthma admission amount evolution process; it can not only reliably reflect the transition situation but also build a bridge between healthcare management and healthcare resource scheduling optimization. For instance, assuming that Markov transition probability between each admission amount state and future distributions of air pollution condition is given, then, future distributions of the admission amount state are also known. Hence, healthcare resource scheduling according to future distributions of the admission amount state will achieve a better performance. Markov models allow the modelling of patient follow-up as a succession of transitions between states over time. They are quantified as the rate of transition and expressed in number of transitions. The model was considered to be homogeneous; that is, the transition forces are independent of time. To construct the elder asthma admission Markov chain, we use the Lorenz curve and OR analysis to determine admission amount states and severity of air pollution. In economics, the Lorenz curve is a graphical representation of the distribution of income or of wealth, while in this situation, the Lorenz curve is a graphical representation of the distribution of daily admission amount. In statistics, the odds ratio (OR) is one of three main ways to quantify how strongly the presence or absence of property A is associated with the presence or absence of property B in a given population; while in our research, OR is employed to quantify the association between environmental properties and admission amount states. Then, a multistate model (MSM) was used to calculate the Markov transition probabilities. All models were fitted using R software (version 3.3.2, R Foundation for Statistical Computing, http://cran.r-project.org/) with the mgcv and msm package.

## 3. Results

Table 1 summarizes the basic statistics for our study. From January 1, 2014, to December 31, 2014, a total of 7503 hospital admissions for asthma were recorded. Among them, elder admissions take 1567 admissions. On average, there were approximately 4 admission counts per day in our study area, females accounting for 61%. Hospital admission for asthma was higher in cold season (842 in total, i.e., 2.3 people per day), compared to warm season (725 in total, i.e., 2 people per day). During the study period, the average of daily concentrations was 72 *μ*g/m^3^ for PM2.5, 116 *μ*g/m^3^ for PM10, 17 *μ*g/m^3^ for SO_2_, and 52 *μ*g/m^3^ for NO_2_.

Generally, PM2.5, PM10, SO_2_, and NO_2_ had moderately high correlation coefficients with each other (Table 2).  Table 3 shows the results from the period lag (lag0 to lag0–5) for the percent increase in hospital admission per 10 *μ*g/m^3^ increase in pollution. Among the above four air pollutants, only SO_2_ was significant when it comes to lag0, lag0-1, and lag0–2. The strongest effects were observed at lag0–2 for PM2.5, PM10, and NO_2_ and lag0-1 for SO_2_. A 10 *μ*g/m^3^ increase in concentration of each air pollutant corresponds to a 0.82 (PM2.5, 95% CI: −0.24, 1.89), 0.5 (PM10, 95% CI: −0.29, 1.3), 7.27 (SO_2_, 95% CI: 1.1, 13.82), and 3.26 (NO_2_, 95% CI: −0.66, 7.33) increase in risk of asthma hospitalization. The presented values are in percentages. For all lags, the effects of PM2.5 were always bigger than those of PM10, which indicates that PM2.5 has a stronger association with asthma hospitalization. All four pollutants showed the pattern that the effect increases with lag firstly and then peaks at a certain lag.

**Figure 1:**
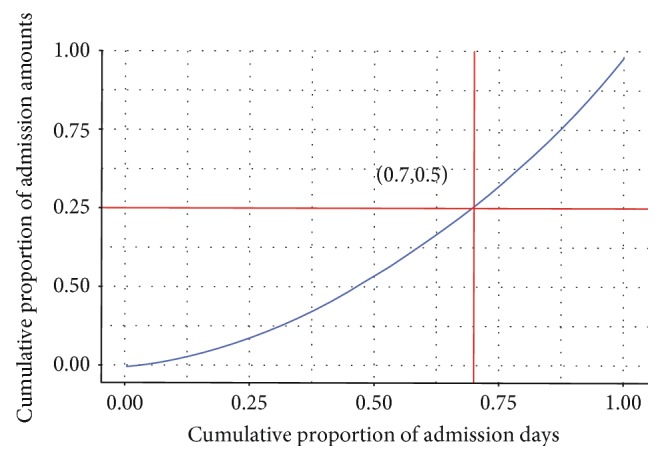
The Lorenz curve of elder asthma admission.

To define different admission amount states, we used the Lorenz curve to analyze the total admission amount.  Figure 1 is the Lorenz curve of elder asthma patient admission, presenting the homogeneous degree of asthma admission for every admission day. The *x*-axis denotes the cumulative proportion of admission days, and the *y*-axis denotes the cumulative proportion of admission counts. The curve shows that the 70% admission days with the lowest admission amount takes 50% admission amount of total admission amount; therefore, the top 30% admission days (the days of which admission amount is not less than 5) are labelled as “high day.”

In the framework of Markov chain, the transition probabilities between different states vary, when decision or situation changes. For instance, when air pollution converts from mild to severe, the transition probability from low-admission amount to high-admission amount increases evidently. In our research, we constructed two transition probability matrices between different states, for mild air pollution and severe air pollution, respectively. Results from Table 3 indicates that air pollution is a risk factor for elder asthma admission; however, which air pollution index is proper to associate with elder asthma admission is still unknown. Therefore, we should construct a meaningful and easy-to-understand index to reflect the Markov transition process. A feasible way is to measure whether within a recent period, air pollution exceeded the concentration threshold not less than certain times. If it is true, the air pollution is severe; otherwise, mild.  Table 4 presents the concentration threshold of the Chinese Ministry of Environmental Protection for each pollutant.

Odds ratio analysis is a vastly used method to select a related index.  Table 5 shows odds ratio between the air pollution index and high elder asthma admission. If the odds ratio is bigger than 1, the independent variable is more likely to be the risk factor; otherwise, it becomes the protective factor. Hence, the index with the biggest odds ratio is considered to be the most appropriate air pollution index. For PM2.5, the appropriate index measures whether within recent 6 days, PM2.5 daily average concentration exceeded the primary standard not less than 6 times. For PM10, the appropriate index measures whether within recent 3 days, PM10 daily average concentration exceeded the primary standard not less than 2 times. For SO_2_, the appropriate index measures whether within recent 2 days, SO_2_ daily average concentration exceeded the primary standard not less than 1 time. For NO_2_, the appropriate index measures whether within recent 6 days, NO_2_ daily average concentration exceeded the primary standard not less than 1 time. Considering that SO_2_ has the biggest odds ratio (4.02, 95% CI: (2.26, 7.18)), severe air pollution is defined as whether within recent 2 days, SO_2_ daily average concentration exceeding 35 *μ*g/m^3^ not less than 1 time.

**Figure 2:**
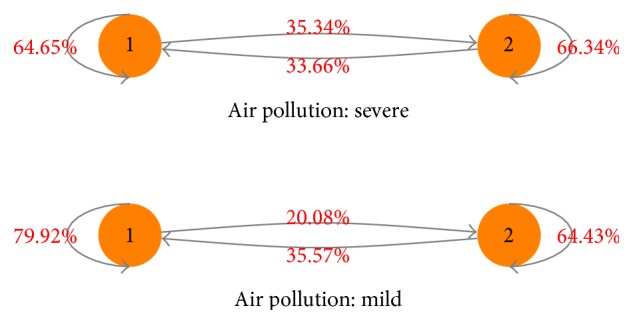
Markov transition probabilities between the high-admission state (state 2) and the low-admission state (state 1).

Figure 2 shows the transition probability between high-admission states and low-admission states under different air pollution situations. When air pollution is severe, the transition probability from a low-admission state (last day) to a high-admission state (next day) is 35.46%, and for the low-admission state (next day), it is 64.54%; the transition probability from a high-admission state (last day) to a low-admission state (next day) is 33.66%, and for the high-admission state (next day), it is 66.34%. While air pollution is mild, the transition probability from a low-admission state (last day) to a high-admission state (next day) is 20.08%, and for the low-admission state (next day), it is 79.92%; the transition probability from a high-admission state (last day) to a low-admission state (next day) is 35.57%, and for the high-admission state (next day), it is 64.43%. These results can be used to construct an asthma resource optimization framework considering air pollution. These transition probabilities also show that, when air pollution gets worse, the transition probability from a low-admission state (last day) to a high-admission state (next day) increases 15.26%, and the transition probability from a high-admission state (last day) to a high-admission state (next day) increases 1.91%.

Table 6 compares the RR among Shanghai, Milan, and this study. For Milan, Santus et al. [23] measured the acute effects between air pollution and elder emergent visit. While admission can be viewed as the aggravation of emergent visit, and admission amount has a tight relation with emergent visit amount. For all four pollutants, the RRs of the above air pollutants in Milan are 3.30 (95% CI: −0.44, 11.70), 3.00 (95% CI: −3.60, 10.10), 9.90 (95% CI: −15.40, 42.80), and 0.80 (95% CI: −4.30, 6.30), respectively, whereas the counterparts in this study are 0.79 (95% CI: −0.23, 1.82), 0.49 (95% CI: −0.27, 1.25), 3.57 (95% CI: 0.55, 6.68), and 3.2 (95% CI: −0.45, 6.98), respectively. The effects of PM2.5, PM10, and SO_2_ in Milan are stronger than those in this study, which may lie in the fact that emergent visits are more sensitive to air pollution. However, the effect of NO_2_ in Milan is much weaker than that in this study; this gap needs further study.

The acute effects of PM10, SO_2_, and NO_2_ are all significant in Shanghai [12]; however, only SO_2_ of those is significant. Besides, the RRs of Shanghai for the three pollutants mentioned above are 1.88 (95% CI: 3.58, 7.35), 4.79 (95% CI: 1.69, 11.27), and 9.38 (95% CI: 3.24, 15.51), respectively, whereas those of this study are 1.44 (95% CI: −2.8, 5.86), 25.81 (95% CI: 4.05, 52.12), and 7.11 (95% CI: −2.5, 17.67), respectively. The results indicate that (1) for PM10 and NO_2_, the effects are slightly weaker than those in Shanghai and (2) for SO_2_, the effects are dramatically stronger than those in Shanghai. These differences may lie when sexual and season factors were not considered.

Table 7 summarizes the estimates of season- and sex-specific effects for each pollutant, with the lags involved in this part being the best lags in Table 3. For PM2.5, the acute effects were not significant when season was not considered. In the male-warm subgroup, the effects were significant and achieved the strongest (10.09), which indicates that PM2.5 matters for male in warm season. PM10 followed the same pattern, and the significant and largest RR is 4.84, which is also smaller than that of PM2.5. However, for SO_2_, all the male subgroups show to be insignificant, whereas the significant effects appeared in cold season or the whole for female subgroups. The largest and significant RR was 13.71 in the female-cold subgroup. NO_2_ also showed no significance in the male subgroup; however, the significance appeared in both cold and warm subgroups. We can find an interesting phenomenon that the male subgroup was only sensitive in warm season, especially for PM2.5 and PM10. For male, the effect was significant only during warm season with PM2.5 and PM10. However, for the female, every effect is significant during cold season. Due to the fact that the strongest effect for each pollutant was in the female-cold subgroup, which indicates that the female-cold subgroup is more fragile to air pollution, we decided to construct the Markov transition probability matrix only for the female-cold subgroup.

**Figure 3:**
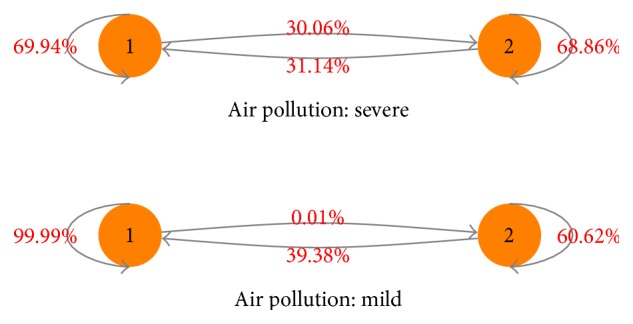
Markov transition probabilities between the high-admission state (state 2) and the low-admission state (state 1) for the female-cold subgroup.

Figure 3 shows the Markov transition probability matrix for the female-cold subgroup, and its corresponding parameters were given in Table 8. When air pollution is severe, the transition probability from a low-admission state (last day) to a high-admission state (next day) is 30.06%, and for the low-admission state (next day), it is 69.94%; the transition probability from a high-admission state (last day) to a low-admission state (next day) is 31.14%, and for the high-admission state (next day), it is 68.86%. However, when air pollution is mild, the transition probability from a low-admission state (last day) to a high-admission state (next day) is 0.01%, and for the low-admission state (next day), it is 99.99%; the transition probability from a high-admission state (last day) to a low-admission state (next day) is 39.38%, and for the high-admission state (next day), it is 60.62%. These results can be used to construct an asthma resource optimization framework considering air pollution. These transition probabilities also show that, when air pollution gets worse, the transition probability from a low-admission state (last day) to a high-admission state (next day) increases 29.07%, and the transition probability from a high-admission state (last day) to a high-admission state (next day) increases 8.24%.

## 4. Discussion

This study certified that air pollutants have adverse short effects on elder hospital admissions for asthma. Such effects were observed for both the gaseous (SO_2_ and NO_2_) and particulate (PM10 and PM2.5) pollutants across all the different sex groups and season groups. PM2.5, PM10, and NO_2_ showed no significant effects on elders, whereas SO_2_ was evidently significant from lag0 to lag0–2.

We also made a comparison with the effects in other studies: (1) When compared with Milan (emergent visit), the effects of PM2.5, PM10, and SO_2_ are stronger than those in this study, which may lie in the fact that emergent visits are more sensitive to air pollution. However, the effect of NO_2_ in Milan is much weaker than that in this study; this gap needs further study. (2) When compared with Shanghai (admission), for PM10 and NO_2_, the effects are slightly weaker than those in Shanghai, whereas for SO_2_, the effects are dramatically stronger than those in Shanghai. These differences may lie when sexual and season factors were not considered.

Sex- and season-specific analysis indicates that for male, the effect was significant only during warm season with PM2.5 and PM10; however, for the female, every effect is significant during cold season.

Precise Markov transition probabilities between high-admission states and low-admission states are obtained by a multistate model. It was also shown that when air pollution gets worse, the transition probabilities from low-admission states and high-admission states to high-admission states increase dramatically. When we focused on the female-cold subgroup, this phenomenon appeared more evidently: the probability increasing due to air pollution worsening of the female-cold subgroup was much dramatic than that of full samples.

When these transition probabilities were combined with the forecast of air pollution, we can obtain the distributions of asthma admission, with reference to asthma healthcare resource demand (such as professional Medicare staff, wards) for a long period. Further, based on these distributions, asthma healthcare resource allocation can be done by the operation research method.

There are three points that should be focused:

1. Among PM2.5, PM10, SO_2_, and NO_2_, only the increment of SO_2_ was significant with that of elder asthma hospitalization. The effects of PM2.5, PM10, and SO_2_ in Milan are stronger than those in this study, which may lie in the fact that emergent visits are more sensitive to air pollution. However, the effect of NO_2_ in Milan is much weaker than that in this study; this gap needs further study.
2. For male, the effect was significant only during warm season with PM2.5 and PM10. However, for the female, every effect is significant during cold season. The strongest effect for each pollutant was in the female-cold subgroup, which indicates that the female-cold subgroup is more fragile to air pollution and that is why we constructed Markov transition probability matrix only for the female-cold subgroup.
3. The difference between full samples and the female-cold subgroup was quite evident: the probability increasing due to air pollution worsening of the female-cold subgroup was much dramatic than that of full samples. That is to say, air pollution matters for the female-cold subgroup more than full samples. This gap may lie when the acute effects between each air pollutant and elder asthma admission vary on sexual and season factors.

## 5. Conclusion

In summary, this study mainly achieved three goals: (1) validating air pollution (PM2.5, PM10, NO_2_, and SO_2_) has a great impact on elder asthma admission. For different air pollution conditions, the index to forecast high admission differs. (2) Outputting an effective air pollution index was performed to associate it with elder asthma admission. (3) Outputting Markov transition probabilities between high-admission states and low-admission states was performed, which could be used to forecast asthma healthcare resource demand when combined with air pollution forecast and then lead to healthcare resource allocation optimization.

Our study has limitations. First, the study design is ecological in nature, which may limit its ability for causal inference. Second, we simply averaged the monitoring results across various stations as the proxy for population exposure level to air pollution, which may raise a number of issues given that pollutant measurements can differ between monitoring locations and that ambient monitoring results differ from personal exposure level to air pollutants. The resulting measurement error may have substantial implication for interpreting time-series air pollution studies. Finally, this research only focuses on elder asthma admission. In fact, asthma outpatient and emergent patient take a large part of asthma healthcare resource. However, we do not have asthma outpatient and emergent patient.

Our work is a novel and fundamental study in asthma resource management. It not only provides a new prospect in the association between air pollution and asthma admission but also leads to a practical framework to implement asthma intervention and to allocate corresponding resource (such as professional Medicare staff, wards). Future work will be done in the aspect of forecasting admission amount, asthma intervention, and corresponding resource allocation.

## Figures and Tables

**Table 1:** Summary statistics of daily asthma hospital admission, air pollutant concentrations, and weather conditions from January 1, 2014, to December 31, 2014.

	*N*	Mean	SD	Min^a^	Q1^a^	Q2^a^	Q3^a^	Max^a^	IQR^a^
All elder	1567	4.29	2.30	0	4	3	6	13	3
Sex									
Male	605	1.66	1.33	0	1	1	2	7	1
Female	962	2.64	1.76	0	2	13	10	2	
Season^b^									
Warm	725	3.96	1.97	0	4	3	5	11	2
Cold	842	4.63	2.55	0	4	36	13	3	
Air pollution concentrations (24 h average)									
PM2.5 (*μ*g/m^3^)	—	72	52	10	38	55	88	396	50
PM10 (*μ*g/m^3^)	—	116	72	20	68	96	147	562	79
SO_2_ (*μ*g/m^3^)	—	17	10	3	11	15	21	61	10
NO_2_ (*μ*g/m^3^)	—	52	16	20	41	50	60	109	19
Meteorological measures									
Min temperature	—	13	7	−2	7	15	20	24	13

PM2.5: particulate matter not greater than 2.5 mm in aerodynamic diameter; PM10: particulate matter not greater than 10 mm in aerodynamic diameter; SO_2_: sulfur dioxide; NO_2_: nitrogen dioxide; ^a^min: minimum; Q1: 25th percentile; Q2: 50th percentile; Q3: 75th percentile; max: maximum; IQR: interquartile range (Q3–Q1). ^b^Cold season: from October to March; warm season: from April to September.

**Table 2:** Pearson correlation coefficients between daily air pollutant concentrations from January 1, 2014, to December 31, 2014.

	PM2.5	PM10	SO_2_	NO_2_
PM2.5	1			
PM10	0.86	1		
SO_2_	0.51	0.53	1	
NO_2_	0.55	0.56	0.47	1

Abbreviations are the same as in Table 1.

**Table 3:** Percent increase (mean and 95% confidence interval) in daily asthma hospital admission associated with a 10 *μ*g/m^3^ increase in air pollutants from January 1, 2014, to December 31, 2014.

	PM2.5	PM10	SO_2_	NO_2_
Lag0	0.54 (−0.44, 1.52)	0.24 (−0.47, 0.95)	6.59 (1.11, 12.36)^∗^	2.4 (−0.87, 5.77)
Lag0-1	0.79 (−0.23, 1.82)	0.49 (−0.27, 1.25)	7.27 (1.1, 13.82)^∗^^§^	3.2 (−0.45, 6.98)
Lag0–2	0.82 (−0.24, 1.89)^§^	0.5 (−0.29, 1.3)^§^	6.94 (0.4, 13.91)^∗^	3.26 (−0.66, 7.33)^§^
Lag0–3	0.74 (−0.35, 1.84)	0.42 (−0.4, 1.24)	5.83 (−0.95, 13.09)	2.69 (−1.44, 6.99)
Lag0–4	0.65 (−0.47, 1.78)	0.34 (−0.5, 1.19)	4.41 (−2.55, 11.87)	2.01 (−2.28, 6.49)
Lag0–5	0.54 (−0.61, 1.7)	0.23 (−0.63, 1.1)	3.12 (−3.97, 10.74)	1.76 (−2.69, 6.41)

Abbreviations are the same as in Table 1. ^∗^*p* < 0.05. ^§^Strongest effect (best lag).

**Table 4:** Concentration threshold of Chinese Ministry of Environmental Protection for each pollutant.

Pollutant	Concentration threshold	
	Primary standard	Second standard
PM2.5	35 *μ*g/m^3^	75 *μ*g/m^3^
PM10	50 *μ*g/m^3^	150 *μ*g/m^3^
SO_2_	50 *μ*g/m^3^	150 *μ*g/m^3^
NO_2_	80 *μ*g/m^3^	—

Abbreviations are the same as in Table 1. — indicates no existence.

**Table 5:** Odds ratio (mean and 95% confidence interval) between the air pollution index and high elder asthma admission.

Odds ratio		Counts of days exceeding the national standard											
		Primary standard						Second standard					
		1	2	3	4	5	6	1	2	3	4	5	6
PM2.5	Lag0	1.61 (1.31, 1.99)	—	—	—	—	—	2.04 (1.82, 2.29)	—	—	—	—	—
	Lag0-1	1.89 (1.26, 2.84)	1.9 (1.61, 2.23)	—	—	—	—	1.84 (1.65, 2.06)	1.9 (1.67, 2.16)	—	—	—	—
	Lag0–2	0.89 (0.56, 1.4)	1.75 (1.37, 2.23)	2.55 (2.18, 2.97)	—	—	—	1.54 (1.37, 1.71)	1.9 (1.69, 2.13)	1.87 (1.61, 2.17)	—	—	—
	Lag0–3	0.41 (0.23, 0.71)	1.13 (0.85, 1.51)	1.89 (1.53, 2.32)	2.7 (2.35, 3.1)	—	—	1.46 (1.31, 1.63)	1.69 (1.51, 1.89)	1.79 (1.57, 2.03)	2.43 (2.06, 2.85)	—	—
	Lag0–4	0.52 (0.15, 1.84)	0.89 (0.64, 1.25)	1.28 (1.02, 1.61)	2.11 (1.74, 2.55)	2.83 (2.49, 3.22)	—	1.27 (1.14, 1.42)	1.52 (1.36, 1.7)	1.7 (1.51, 1.92)	2.05 (1.79, 2.35)	2.88 (2.4, 3.47)	—
	Lag0–5	0.26 (0.05, 1.38)	0.72 (0.45, 1.16)	1.25 (0.94, 1.65)	1.66 (1.32, 2.07)	2 (1.69, 2.37)	3.03 (2.69, 3.43)	1.2 (1.07, 1.35)	1.33 (1.19, 1.48)	1.58 (1.41, 1.77)	2.04 (1.8, 2.31)	2.53 (2.17, 2.95)	3.32^§^(2.71, 4.06)
	Lag0	3.13 (1.67, 5.86)	—	—	—	—	—	1.69 (1.48, 1.93)	—	—	—	—	—

PM10	Lag0-1	NA	1.82 (1.39, 2.38)	—	—	—	—	1.86 (1.65, 2.09)	1.37 (1.16, 1.63)	—	—	—	—
	Lag0–2	NA	3.34^§^ (1.33, 8.38)	1.99 (1.59, 2.48)	—	—	—	1.63 (1.45, 1.82)	1.69 (1.47, 1.94)	1.81 (1.48, 2.21)	—	—	—
	Lag0–3	NA	2.68 (0.43, 16.84)	1.89 (1.26, 2.84)	2.65 (2.14, 3.3)	—	—	1.83 (1.64, 2.05)	1.54 (1.35, 1.74)	2.01 (1.71, 2.37)	2.07 (1.63, 2.62)	—	—
	Lag0–4	NA	NA	1.33 (0.67, 2.64)	1.77 (1.31, 2.39)	2.33 (1.95, 2.79)	—	1.69 (1.51, 1.89)	1.51 (1.34, 1.7)	2.07 (1.8, 2.39)	2.14 (1.77, 2.58)	3.19 (2.43, 4.18)	—
	Lag0–5	NA	NA	1.59 (0.23, 11.13)	1.63 (0.98, 2.71)	1.86 (1.42, 2.44)	2.1 (1.8, 2.46)	1.61 (1.44, 1.8)	1.49 (1.33, 1.67)	2.1 (1.84, 2.39)	2.16 (1.84, 2.52)	2.97 (2.39, 3.7)	3.2 (2.32, 4.42)
	Lag0	3.9 (1.27, 12.01)	—	—	—	—	—	NA	—	—	—	—	—

SO_2_	Lag0-1	4.02^§^ (2.26, 7.17)	NA	—	—	—	—	NA	NA	—	—	—	—
	Lag0–2	3.27 (2.19, 4.88)	NA	NA	—	—	—	NA	NA	NA	—	—	—
	Lag0–3	2.96 (2.17, 4.05)	NA	NA	NA	—	—	NA	NA	NA	NA	—	—
	Lag0–4	2.81 (2.17, 3.65)	NA	NA	NA	NA	—	NA	NA	NA	NA	NA	—
	Lag0–5	3.13 (2.51, 3.9)	NA	NA	NA	NA	NA	NA	NA	NA	NA	NA	NA
	Lag0	3.2 (2.32, 4.42)	—	—	—	—	—						

NO_2_	Lag0-1	2.97 (2.39, 3.7)	1.65 (0.73, 3.72)	—	—	—	—						
	Lag0–2	2.87 (2.4, 3.42)	2.82 (1.84, 4.33)	1.92 (0.54, 6.79)	—	—	—						
	Lag0–3	2.44 (2.09, 2.86)	3.58 (2.63, 4.86)	2.23 (1.14, 4.38)	NA	—	—						
	Lag0–4	2.37 (2.06, 2.73)	3.51 (2.7, 4.55)	3.16 (1.8, 5.18)	0.95 (0.12, 7.63)	NA	—						
	Lag0–5	2.04 (1.78, 2.33)	3.77 (3.02, 4.71)	3.89^§^(2.83, 5.34)	2.61 (1.28, 5.33)	NA	NA						

Abbreviations are the same as in Table 1. ^§^The biggest OR. — indicates no existence. NA means cannot be calculated.

**Table 6:** Comparison of RR among Shanghai, Milan, and this study.

		PM2.5	PM10	SO_2_	NO_2_
Shanghai (admission) [12]	Lag (increment)	—	Lag0-1 (60 *μ*g/m^3^)	Lag0-1 (36 *μ*g/m^3^)	Lag0-1 (29 *μ*g/m^3^)
	RR (95% CI)	—	1.88 (3.58, 7.35)^∗^	4.79 (1.69, 11.27)^∗^	9.38 (3.24, 15.51)^∗^

This study (admission)	Lag (increment)	—	Lag0-1 (60 *μ*g/m^3^)	Lag0-1 (36 *μ*g/m^3^)	Lag0-1 (29 *μ*g/m^3^)
	RR (95% CI)	—	1.44 (−2.8, 5.86)	25.81 (4.05, 52.12)^∗^	7.11 (−2.5, 17.67)

Milan (emergent visit) [23]	Lag (increment)	Lag0–2 (10 *μ*g/m^3^)	Lag0–2 (10 *μ*g/m^3^)	Lag0–2 (5 *μ*g/m^3^)	Lag0–2 (10 *μ*g/m^3^)
	RR (95% CI)	3.30 (−4.40, 11.70)	3.00(−3.60, 10.10)	9.90(−15.40, 42.80)	0.80(−4.30, 6.30)

This study (admission)	Lag (increment)	Lag0-1 (10 *μ*g/m^3^)	Lag0-1 (5 *μ*g/m^3^)	Lag0-1 (10 *μ*g/m^3^)	Lag0-1 (10 *μ*g/m^3^)
	RR (95% CI)	0.79 (−0.23, 1.82)	0.49 (−0.27, 1.25)	3.57 (0.55, 6.68)^∗^	3.2 (−0.45, 6.98)

Abbreviations are the same as in Table 1. — indicates not mentioned. ^∗^*p* < 0.05.

**Table 7:** Percent increase (mean and 95% confidence interval) in asthma hospital admission associated with a 10 *μ*g/m^3^ increase in air pollutant concentrations by season and sex.

Sex	Season	PM2.5 (lag0–2)	PM10 (lag0–2)	SO_2_ (lag0-1)	NO_2_ (lag0–2)
Both	Both	0.82 (−0.24, 1.89)	0.5 (−0.29, 1.3)	7.27 (1.1, 13.82)^∗^	3.2 (−0.45, 6.98)
	Warm	4.72 (2.26, 7.23)^∗^	2.48 (0.98, 4.01)^∗^	3.53 (−8.6, 17.26)	−7.73 (−12.98, −2.16)^∗^
	Cold	0.94 (0.15, 1.74)^∗^	0.93 (0.31, 1.55)^∗^	9.18 (4.27, 14.32)^∗^	7.39 (4.26, 10.62)^∗∗^

Male	Both	−0.13 (−1.83, 1.59)	−0.12 (−1.39, 1.16)	2.95 (−6.47, 13.31)	0.77 (−5.34, 7.28)
	Warm	10.09 (6, 14.33)^∗∗^	4.84 (2.41, 7.33)^∗^	12.31 (−7.64, 36.56)	−1.06 (−9.79, 8.51)
	Cold	−0.02 (−1.26, 1.24)	0.28 (−0.7, 1.26)	1.95 (−5.07, 9.5)	3.28 (−1.4, 8.18)

Female	Both	1.38 (0.03, 2.75)	0.88 (−0.12, 1.89)	9.93 (1.95, 18.54)^∗^	4.71 (−0.31, 9.99)
	Warm	0.92 (−2.3, 4.25)	0.74 (−1.26, 2.78)	2.36 (−13.31, 20.85)	−11.39 (−18.04, −4.19)^∗^
	Cold	1.5 (0.44, 2.57)^∗^	1.32 (0.49, 2.16)^∗^	13.71 (6.87, 20.99)^∗^	9.31 (5.04, 13.76)^∗∗^

Abbreviations are the same as in Table 1. Cold season: from October to March; warm season: from April to September. ^∗^*p* < 0.05; ^∗∗^*p* < 0.01.

**Table 8:** Corresponding parameters of the female-cold subgroup.

Subgroup	A1	A2	A3	A4	A5	A6
Female-cold	PM2.5	Lag0–4	35 *μ*g/m^3^	4 days	2 persons per day	15.11 (2.72, 83.78)

A1: pollutant; A2: lag; A3: concentration threshold; A4: counts exceeding the concentration threshold; A5: admission amount threshold; A6: OR.

## Abbreviations

PM2.5: Particulate matter not greater than 2.5 mm in aerodynamic diameter
PM10: Particulate matter not greater than 10 mm in aerodynamic diameter
SO_2_: Sulfur dioxide
NO_2_: Nitrogen dioxide
QALY: Quality-adjusted life years.

## Additional Points

*Availability of Data and Materials*. The data supporting the study findings will not be shared since it is an organizational property. Data was anonymous, and study subjects could not be identified.
